# Introduction to the Sixth Global Summit on the Health Effects of Yogurt: Yogurt, More than the Sum of Its Parts

**DOI:** 10.1093/advances/nmz017

**Published:** 2019-09-13

**Authors:** Sharon M Donovan, Olivier Goulet

**Affiliations:** 1 Food Science and Human Nutrition, University of Illinois, Urbana, IL; 2 Department of Pediatric Gastroenterology-Hepatology-Nutrition, National Reference Center for Rare Digestive Disease, Hôpital Necker-Enfants Malades, University of Paris Descartes, Paris, France

**Keywords:** yogurt, food matrix/dairy fat, fermented dairy, type 2 diabetes, cardiovascular disease

## Abstract

Foods are not only a collection of individual components but are complex matrices. The food matrix is defined by the USDA as “the nutrient and nonnutrient components of foods and their molecular relations.” The matrix of a food is an important factor in evaluating its nutritional and health contributions to the consumer. Dairy foods are a complex mix of various nutrients and other components, which together form the food matrix. There are three main types of dairy food matrices: liquid (milk, some fermented milks), semi-solid (yogurt, some fresh cheeses), and solid (most cheeses). The nutritional value of dairy foods is determined by their nutrient composition and matrix structure, which can affect digestibility and the bioavailability of nutrients. Additionally, a number of studies have shown that the health effects of dairy products, of similar nutrient content, vary by their matrix.

## Introduction

Foods are not only a collection of individual components but are complex matrices. The food matrix is defined by the USDA as “the nutrient and nonnutrient components of foods and their molecular relations” (1). The matrix of a food is an important factor in evaluating its nutritional and health contributions to the consumer (2). Dairy foods are a complex mix of various nutrients and other components, which together form the food matrix (3). There are three main types of dairy food matrices: liquid (milk, some fermented milks), semi-solid (yogurt, some fresh cheeses), and solid (most cheeses) (3). The nutritional value of dairy foods is determined by their nutrient composition and matrix structure, which can affect digestibility and the bioavailability of nutrients (2, 3). Additionally, a number of studies have shown that the health effects of dairy products, of similar nutrient content, vary by their matrix (4–7).

Yogurt is a nutrient-dense food that is an excellent source of high-quality protein and calcium, as well as other minerals (iodine, magnesium, potassium, phosphorus) and vitamins (pantothenic acid (B_5_), riboflavin (B_2_), vitamin B_12,_ vitamins A and D), if supplemented to the dairy starting material (8). Thus, yogurt can be an important dietary source of a number of shortfall nutrients (9). Indeed, studies have shown that yogurt consumption is associated with better diet quality in both children (10) and adults (11).

To add further complexity, yogurt is a fermented food. Although the milk starting materials and ferments used to produce yogurt can vary across the globe (12), in order to meet the Codex Alimentarius standard, yogurt must contain two strains of live bacteria, *Lactobacillus delbrueckii* subsp *bulgaricus* and *Streptococcus thermophilus*, at a content of ≥ 10 million bacteria/g in the final product (13). The metabolic activity of microbes result in the production of B vitamins, conjugated linoleic acid, γ-aminobutyric acid, peptides, and volatile fatty acids (14–16).

The consumption of fermented foods, such as yogurt and other cultured dairy products, has been shown to modify the composition of the microbiome, and some of the health benefits of yogurt, such as in obesity, type 2 diabetes, and CVDs, are proposed to be mediated through microbiome modulation (15, 17–19). Thus, it is likely that the health benefits of yogurt are derived from its nutrient composition, probiotic bacteria, and products of fermentation. As such, the matrix of yogurt is distinct from other nonfermented dairy products and its associated health benefits appear to be more complex than the sum of its parts (nutrients, live microbes, fermentation products).

Accordingly, the goals of the Sixth Global Summit on the Health Effects of Yogurt, held at Nutrition 2018 hosted by the American Society for Nutrition, were to: *1*) review the role of the whole matrix of yogurt versus single nutrients on health; *2*) summarize the research on the effects of fermented foods on human health; *3*) describe the potential role of bioactive peptides produced during yogurt fermentation on cardiometabolic health; and *4*) consider the matrix in moderating the effects of low- and full-fat dairy on health outcomes.

In the first presentation, Dariush Mozaffarian described how, over time, the focus on preventing and alleviating nutrient deficiencies led the field of nutrition to take the reductionist approach of considering individual nutrients and using that information to form dietary recommendations (11). In contrast, he recommended that dietary recommendations should focus on foods rather than nutrients. Although the 2015 Dietary Guidelines for Americans placed a greater focus on healthy eating patterns compared with previous guidelines, the guidelines still recommended that the public “choose lower fat versions of milk, yogurt, and cheese in place of whole milk products and regular cheese” (12) as a strategy for reducing saturated fat intake. However, current clinical and epidemiological evidence suggests that different food sources of saturated fats have varied effects on health in terms of obesity and cardiometabolic outcomes, including type 2 diabetes, and thus, all saturated fats should not be treated the same in dietary recommendations (11). For example, results from a 14-y follow-up of over 26,000 individuals in the Malmö Diet and Cancer cohort demonstrated that a high intake of high-fat, but not low-fat dairy products, decreased the risk of type 2 diabetes (9). Additionally, saturated fat from meat was significantly associated with a higher risk of metabolic disease, but not saturated fat from dairy. The effect of the dairy matrix in mediating the lipid response to the daily consumption of 40 g dairy fat was previously shown in a 6-wk randomized controlled trial (10). They observed that dairy fat in the form of cheese reduced total cholesterol and low-density lipoprotein-associated cholesterol compared with the same constituents eaten in different matrices (butter supplements with calcium caseinate powder and a calcium supplement) (10). Mozaffarian concluded that the “current science supports eating more dairy and especially yogurt, and whether low-fat or full-fat dairy products are consumed should be left to personal preference” (11).

Robert Hutkins and Andre Marette have explored the potential health benefits of the microbial components of yogurt. Hutkins reviewed the properties of yogurt cultivars and physiological and ecological challenges faced by fermentation- and other food-associated microbes during digestion and transit through the gastrointestinal tract and the evidence that many of these organisms can survive digestion (15). In addition to the yogurt starter culture organisms (*Lactobacillus delbrueckii* subsp. *bulgaricus* and *Streptococcus thermophilus*), other strains of *Lactobacillus* and *Bifidobacterium* species may be added to yogurt due to their probiotic activities. Hutkins then reviewed the epidemiological evidence for the health benefits associated with the consumption of fermented foods, including yogurt, and the evidence that the microorganisms present in yogurt may contribute to these health benefits (15). Potential mechanisms of action underlying the health benefits of yogurt, including lactose fermentation, production of nutrients, and inactivation of antinutrients were discussed, and potential mechanisms whereby the microbes and their fermentation products could interact with the host epithelium and immune system were summarized. He concluded that diets rich in fermented foods can contribute significant numbers of food-associated microbes to the gastrointestinal tract and have the potential to influence the function of the gut microbiota and improve host health (15).

In the past, little attention has been focused on the peptides produced from proteins during the microbial fermentation of foods. André Marette began by reviewing the scientific evidence linking microbial dysbiosis and cardiometabolic diseases and potential underlying mechanisms of action (14). He then presented the concept that bioactive peptides generated from dairy proteins (casein and whey) have the potential to exert biological effects within the digestive tract and in extra-intestinal tissues, such as the vascular, nervous, immune, and endocrine systems, with the net effect of reducing the risk of cardiometabolic diseases (20). He presented results of ongoing work in his laboratory testing the effect of skim milk powder fermented using yogurt cultivars (yogurt) versus *Lactobacillus helveticus* (fermented milk). He demonstrated that low molecular weight (MW) peptides were produced in both the yogurt and fermented milk, but a higher percentage of peptides < 2,000 MW were present in the yogurt (46%) compared with fermented milk (33%). Upon incorporating the different protein preparations in high-fat/high-sucrose diets fed to mice, the different types of dairy products exerted specific effects on glucose and lipid metabolism, with fermented dairy products generally producing greater metabolic and anti-inflammatory effects than the skim milk powder (15). Ongoing work is testing whether peptides released during fermentation can explain some of the cardiometabolic health benefits associated with yogurt consumption.

In the final presentation, Arné Astrup revisited the concept that differences in dietary matrices between meat and dairy, particularly yogurt, are important when considering recommendations to reduce saturated fat intake (8). Most current public health recommendations support reducing overall fat, particularly saturated fat consumption, but do not differentiate between the dietary sources (2, 21). Astrup reviewed the accumulating data from prospective cohort studies and randomized trials over the last 10 y showing no significant evidence associating dietary saturated fat intake with an increased risk of coronary artery disease or CVD (8). When focusing more specifically on dairy intake and low- versus high-fat dairy, dairy intake is typically associated with a lower risk of coronary artery disease, CVD, and diabetes with no difference between low- and high-fat options (8). He concluded with a statement issued by an expert workshop held in Gentofte, Denmark in 2016, which stated that “current evidence does not support a positive association between the intake of dairy products and risk of CVD (e.g., stroke and coronary artery disease) and type 2 diabetes” (6). The consensus also concluded that “different dairy structures and common processing methods may enhance interactions between nutrients in the dairy matrix, which may modify the metabolic effects of dairy consumption” (6).

In conclusion, a robust body of epidemiological observations, and a growing body of evidence emerging from randomized controlled trials, support the viewpoint that consumption of fermented dairy products can provide a wide range of health benefits (22). The unique matrix of yogurt relative to nonfermented dairy products including the presence of live bacteria and their fermentation products, including bioactive peptides, are thought to play a role in the health benefits associated with yogurt consumption (10). Additionally, epidemiological evidence supports the conclusion that saturated fat within the dairy matrix imparts different health effects than saturated fat within the matrix of meat, particularly processed meat (23). Astrup concluded “considering the evidence, daily consumption of both full-fat and fat-reduced yogurt and other fermented products should be encouraged as part of a balanced diet to reduce the risk of chronic diseases. Selecting a single nutrient, such as saturated fat, and assigning blame for overall health effects is shortsighted. There is, however, a need for mechanistic studies to provide plausible biologic explanations for the beneficial effects of dairy on the risk of CVD and type 2 diabetes” (8). Ultimately, the inclusion of fermented dairy products in food-based dietary guidelines, not only as a dairy option, but as a significant source of viable bacteria and fermentation products, could contribute to improved public health and should be considered (24).

## Notes

This article appears as part of the supplement “Yogurt, more than the sum of its parts – Sixth Global Summit on the Health Effects of Yogurt Proceedings” sponsored by Danone Institutes International. The guest editor of the supplement has the following conflict of interest: SMD co-chairs the Yogurt in Nutrition Initiative. She received reimbursement for travel expenses and an honorarium from Danone Institutes International for chairing the Sixth International Yogurt in Nutrition Summit at the Nutrition 2018 meeting in June 2018 in Boston, MA. Publication costs for this supplement were defrayed in part by the payment of page charges. The opinions expressed in this publication are those of the authors and are not attributable to the sponsors or the publisher, Editor, or Editorial Board of *Advances in Nutrition*.

Author disclosures: SMD and OG are co-chairs of the Yogurt in Nutrition Initiative. Travel expenses and an honorarium were paid to SMD and OG by Danone Institutes International for chairing the Sixth International Yogurt in Nutrition Summit at the Nutrition 2018 meeting in June 2018 in Boston, MA. OG is also the Chair of Danone Institutes International.
